# Kidneys for Sale? A Commentary on Moeindarbari’s and Feizi’s Study on the Iranian Model

**DOI:** 10.3389/ti.2022.10530

**Published:** 2022-06-24

**Authors:** Frederike Ambagtsheer, Sean Columb, Meteb M. AlBugami, Ninoslav Ivanovski

**Affiliations:** ^1^ Department of Internal Medicine, Erasmus MC Transplant Institute, University Medical Center Rotterdam, Rotterdam, Netherlands; ^2^ School of Law and Social Justice, The Liverpool Law School, University of Liverpool, Liverpool, United Kingdom; ^3^ Multi-Organ Transplantation Center, King Fahad Specialist Hospital-Dammam, Dammam, Saudi Arabia; ^4^ Clinical Hospital Zan Mitrev, University “Sts Cyril and Methodius”, Skopje, North Macedonia

**Keywords:** ethics, organ trafficking, government regulation, organ trade, payments

Over the last years, efforts by transplant professionals and transplant organizations have resulted in the strengthening of laws and sentences against virtually all forms of organ trade (1–4). The prevailing belief is that organ trade can be prevented by countries becoming “self-sufficient” (4, 5). Iran is the only country that reports to have eliminated its kidney transplant wait list (6, 7). Yet, it is largely condemned for having accomplished this by paying living kidney donors (8–10). Transplant professionals from Iran state that they are often prevented from presenting data about the Iranian model at international transplant conferences and in transplant journals. Furthermore, the regulations that underlie Iran’s decentralized, semi-regulated organ payment programs, differ between the country’s states, leading to differing outcomes (11–14). These cross-country variations, in conjunction with the limited available data, hampers an in-depth understanding of the Iranian model (10, 15, 16).^1^

Moeindarbari’s and Feizi’s study contributes to vital knowledge gaps in this regard. Drawing on a unique data-set collected from the Kidney Foundation in Mashhad, Moeindarbari and Feizi present an analysis of price arrangements between 436 donors and recipients. The findings illustrate, amongst other things, the effects of education, gender, age difference and donor-recipient relationships on kidney prices. In addition, the findings suggest that related donors sell their kidneys to close relatives for a significantly lower price. Government payments are additionally made under the scrutiny of the Ministry of Health for all transplant-related expenses. The authors further explain that donors are provided with medical coverage for 1 year after the nephrectomy and that they are exempted from military service (6).

There are however some concerns about the Iranian model. Mashhad’s kidney transplant program tolerates side payments between recipients and donors besides the fixed government fee. This is problematic because prices fluctuate according to the bargaining skills and abilities of donors and recipients. These unregulated transactions in turn may cause and exacerbate a variety of issues including inequality and interpersonal exploitation. Furthermore, while donors are provided with medical coverage for 1-year post-donation, it is unclear whether life-long follow up is guaranteed.

Moeindarbari and Feizi recognize these concerns and state that a monopsonistic program, where the government pays a fixed sum to donors and where patients do not pay, would allow for more equality and fairness (6). Although a monopsonistic transplant program would not address the conditions of poverty that compel people to sell a kidney, it could reduce the risk of interpersonal exploitation by preventing donors and recipients from negotiating payments (17, 18). While we oppose Iran’s tolerance of unregulated organ payments between donors and recipients, removing criminal penalties for selling a kidney at the very least enables kidney sellers to report harm without risking prosecution (19). Previous research from Iran (13, 20), and from Mashhad in particular (11, 16), suggests that the degree of exploitation reported by Iranian kidney donors is less severe than those who sell their kidneys on the black market, because Iranian kidney donors are protected by law (11, 16). Moeindarbari and Feizi corroborate these findings by pointing out that medical teams in Mashhad have no share of the money paid by the recipient to the donor, that prospective donors are informed about the potential health consequences of their donation and that they receive pre –and post-operative care (6). Any examination of the Iranian model should thus compare the well-being of its donors to those who sell their kidneys on the black market (16, 17, 21).

A growing body of empirical evidence from a number of countries reveals that while organ sales are prohibited by law, they are tolerated in practice (19, 22-26). In addition, research assessing the impact of prohibitive measures suggests that organ trade is being pushed further underground, increasing the role of criminal intermediaries, and exposing donors to more violent means of recruitment (19, 27). Studies further indicate that transplant professionals who facilitate illegal transplants can also be complicit in the exploitation of donors and recipients by not providing (adequate) pre –and post-operative care (29–32). There is however a critical lack of attention for the implications of prohibition and a lack of accountability of those who facilitate illegal transplants, including medical institutions and medical staff (19, 28, 29). Although complicit transplant professionals reportedly profit the most from illegal transplants (19, 29, 32), successful convictions of medical institutions and their staff remain virtually absent (22, 29, 32, 33). The reluctance of organ sellers to report harm (because they risk conviction), further inhibits investigation and prosecution of criminal cases (19, 29).

More empirical data is needed to develop workable solutions grounded in the empirical reality of people directly affected by the trade in organs. Dismissing evidence-based studies assessing the impact of regulatory controls in Iran, currently the only country with a semi-regulated organ market, would be counterintuitive. The implications of prohibition and the growing organ scarcity warrant a data-driven exploration of alternative models that move beyond prohibition and that may more effectively reduce the risk of exploitation of vulnerable donors and diminish patient mortality on transplant wait lists (19, 28, 34).

To this end, more rigorous data from Iran is needed that demonstrates how exactly its organ payment schemes reduce the risk of exploitation. It would be particularly helpful to learn more about donors’ and recipients’ experiences with and attitudes towards Iran’s organ payment programs (11). While Moeindarbari’s and Feizi’s analysis is perhaps more useful for economists who study market designs, studies about Iran’s organ payment programs should not be rejected exclusively on moral grounds. Rather, an honest and open dialogue is needed in which data from different countries and models is comparatively discussed. To this end, studies from Iran, even if we disagree with them, should be welcomed.

## Footnotes

1 In fact this raises the question whether “the Iranian Model” is an appropriate term. The term, “Iranian models,” seems more suitable.
